# Long-term Follow-up of Bilateral Cleft Lip and Palate: Incidence of Speech-Correcting Surgeries and Fistula Formation

**DOI:** 10.1177/10556656221102816

**Published:** 2022-06-20

**Authors:** Charlotta Gustafsson, Arja Heliövaara, Jorma Rautio, Junnu Leikola

**Affiliations:** Cleft and Craniofacial Center, Helsinki University Hospital, Helsinki, Finland

**Keywords:** velopharyngeal function, palatoplasty, surgical technique, bone grafting

## Abstract

**Introduction:**

While bilateral cleft lip and palate (BCLP) constitutes a clinical challenge for the whole cleft team, the ideal surgical protocol remains obscure. This study presents the long-term burden of care in terms of secondary surgeries, defined as fistula repair and speech-correcting surgeries (SCS), in a single center. Outcomes of two surgical protocols utilized over the years were also compared.

**Material and Methods:**

A retrospective single-center analysis of 81 non-syndromic children with complete BCLP born between 1990 and 2010. Two surgical protocols comprising single-stage and two-stage (delayed hard palate closure) procedures were compared. Outcome was analyzed at the time of alveolar bone grafting (ABG) and post-ABG.

**Results:**

Altogether 54 children (66.7%) had underwent secondary surgery by the time of bilateral ABG. At this point, 38.3% (n = 31) of patients had received SCS and 49.4% (n = 40) had undergone fistula repair. The corresponding incidences at the end of follow-up were 46.9% (n = 38) and 53.1% (n = 43). No significant difference emerged in SCS incidence between the 2 protocols; however, prior to ABG the single-stage protocol had a significantly lower need for fistula repair. Regarding the location of fistulas, some differences were observed, with the single-stage procedure more associated with anterior fistulas.

**Conclusion:**

BCLP has a high surgical burden of care in terms of secondary surgeries, defined as SCS and fistula repair. In our experience, the single-stage protocol, particularly the two-flap technique, offers better results in the management of BCLP than the two-stage approach with a short delay in hard palate closure.

## Introduction

The deviant and unique anatomy of bilateral cleft lip and palate (BCLP) contributes to obvious surgical challenges during primary cleft repair.^1,2^ Dehiscence, such as palatal fistulas, is a well-known and often challenging complication following primary repair. Especially patients with BCLP are prone to develop fistulas due to the extensive nature of the cleft and the inevitable tension occurring at repair. A short palate, characteristic of patients with corrected cleft palates, may further predispose to speech-related problems and velopharyngeal insufficiency (VPI). These undesirable factors might have a detrimental impact on the surgical results and the growing child, often requiring secondary operations.^3,4^

As the optimal surgical treatment protocol regarding timing and technique utilized for cleft palate repair remains open, the techniques have evolved by small modifications to existing methods and variations in the timing of repair. Although the general surgical principles and techniques for BCLP are similar to those applied in the repair of unilateral cleft lip and palate (UCLP), the optimal surgical protocol for BCLP is unclear. Given the scarcity of this cleft type, few, if any, institutions have sufficiently large caseloads to enable studies to investigate this properly, necessitating large multicenter studies. Thus, no universal standardized treatment protocols exist and great diversity among treatment protocols is present between centers.

In the literature, the surgical burden of care and long-term outcomes are often underreported, while the surgical outcomes in cleft populations are typically measured by speech-related outcomes and fistula prevalence. In this study, we aimed to investigate the long-term burden of care in terms of secondary surgeries, defined as speech-correcting surgeries (SCS) and fistula repair rates, as a quality measurement of primary surgery in a non-syndromic BCLP population at a single cleft center. Our secondary outcome was to examine the impact of two surgical protocols utilized over the years on the secondary surgery outcomes.

## Methods

### Study Design and Patients

The study was conducted as a retrospective, single-center observational analysis of collected records of non-syndromic children with complete BCLP, including Simonart's bands with complete alveolar clefts, treated at the Cleft and Craniofacial Center, Department of Plastic surgery at Helsinki University Hospital, Finland. The study protocol was approved by Helsinki University Hospital, and the principles outlined in the Declaration of Helsinki were followed. The children were born between 1990 and 2010. Patients with syndromes, other craniofacial anomalies in the head or neck region, incomplete cleft, or inadequate data were excluded.

The study's primary purpose was to determine the long-term incidence of SCS and repair rates for postalveolar fistulas in a BCLP population. The secondary objective was to compare 2 different surgical protocols utilized over the past decades at the cleft center. Data were analyzed at the time of the bilateral secondary alveolar bone grafting (ABG), with cancellous bone graft from the iliac crest, which is usually performed prior to the eruption of the upper canines, between 9 and 11 years of age. Moreover, to receive a broader long-term picture regarding the need for secondary surgery, the outcomes were also analyzed at the end of patients’ total follow-up or at data collection (February 2021) after receiving ABG (post-ABG).

### Classification of Primary Surgery

The surgical protocols for BCLP—and for UCLP described by the Author^5^—have varied over the past decades at the cleft center. Mainly, the surgical procedures have followed the same principles as for UCLP in both single-stage and two-stage procedures, however, some disparities have occurred throughout the years.

Initially, our primary method for lip repair was staged without presurgical orthopedics; lip adhesion at 2 months, with the aim of setting back the premaxilla, while the complete cheiloplasty, was performed in separate operations at 6 months and 8 months of age according to the Milliard^6^ method along with anterior vomerplasty. However, often when performing the second lip repair the cleft in the anterior hard palate was so narrow that closure with a vomer flap was not feasible. Simultaneously, nose repair was performed with limited mobilization of the alar cartilages and an attempt to fix them with bolster sutures which were removed 3 to 4 days postoperatively. Along with the aforementioned protocol for lip and nose repair, the standard technique for palate repair was up to the 1990s, the Veau-Wardill-Kilner (V-W-K)^7^ single-stage technique performed at 12 months of age.

Since 1992, nasal repair has taken place in combination with primary repair of the lip, without presurgical nasoalveolar molding or infant orthopedics prior to surgery. Lip adhesion was considered an unnecessary additional procedure often associated with scarring, making the final cheiloplasty more difficult; it was therefore discarded, as was anterior vomerplasty. The lip repair protocol was further changed to a single-stage “straight-line” bilateral repair at 3 to 4 months. Here, the muscle were sutured in the midline (tension was occasionally unavoidable), discarding the vermilion of the prolabium as the reconstruction of the vermilion was performed with lateral mucosal flaps, thereby representing a modified technique to the principles described by Garcia-Velasco and Nahás.^8^ At the same time, the nose repair was performed, by freeing the alar cartilages and fixing them with buried resorbable mattress monofilament sutures attempting to lengthen the columella and rotating the cartilages medially and anteriorly into a more anatomical position. The cleft palate repair was performed at 10 to 12 months of age, according to the two-flap technique described by Bardach^1^ in combination with a vomer flap. However, for less extensive clefts the minimal incision technique^9^ was utilized, and lateral relaxing incisions were applied when needed.^10^

During enrollment of the patients in the Scandcleft study (SC) (1997-2006)^11^ a two-stage cleft palate repair was also employed in the BCLP population, with early soft palate closure combined with lip repair at 4 months of age, while hard palate closure was performed with a short delay at 12 months of age. While some patients with BCLP received repair by the previously described protocol, the single-stage protocol was still utilized at the center. However, after the SC study the two-stage protocol with early vomer flap in combination with lip repair at 4 months and delayed soft palate closure 6 months later was introduced at our center, which has since remained the standard protocol.

### Classification of SCS

The patient's speech was frequently evaluated at follow-up visits by the cleft team and an experienced speech pathologist according to the standard treatment protocol at the cleft center. Speech evaluation followed a 5-point numeric scale described earlier.^12^ Children presenting with moderate-to-severe VPI or insufficient improvement after speech therapy were recommended to undergo SCS (VPI surgery). For speech-improving surgery, Furlow Z-plasty^13^and superiorly based pharyngeal flaps were employed. However, for the past 20 years, the Furlow-Z-plasty has been utilized as the primary SCS procedure of choice regardless of the VPI severity or the velopharyngeal gapsize. In our opinion this method is often more suitable than pharyngeal flaps, particularly considering the postoperative recovery and less risk for airway obstruction, especially in small children. If VPI is not adequately corrected by the Furlow-Z-plasty, a secondary SCS procedure is performed by a pharyngeal flap.

### Classification of Fistulas

**Figure 1.**
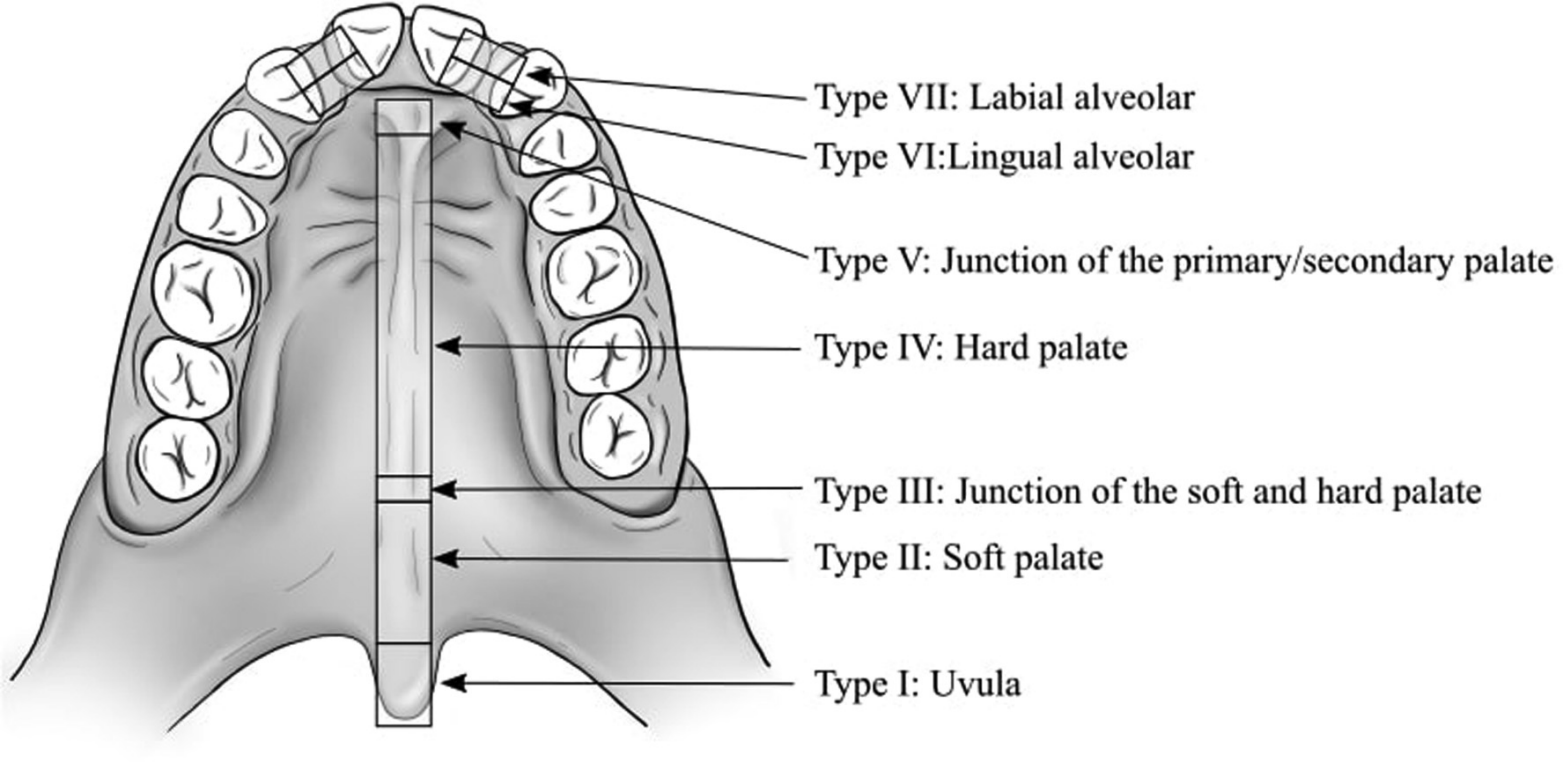
Pittsburgh fistula classification system originally described by Smith et al.^14^

A fistula was considered an unintended defect in the corrected palate. Due to the retrospective nature of the study and the lack of consistent methodologies and reporting standards for palatal fistulas, only fistulas that had received a clinical recommendation for surgical closure were counted. The operated fistulas were identified from the surgical records and localized through the Pittsburgh classification system^14^ (Figure 1). Only postalveolar defects were counted (Pittsburgh I-V) as an unintended palatal fistula that occurred as a result of improper healing of the palate. Several fistulas in the palate were counted as separate fistulas; however, if a fistula crossed several locations, it only counted as one defect, defined according to its most anterior position (Figure 1).

**Figure 2.**
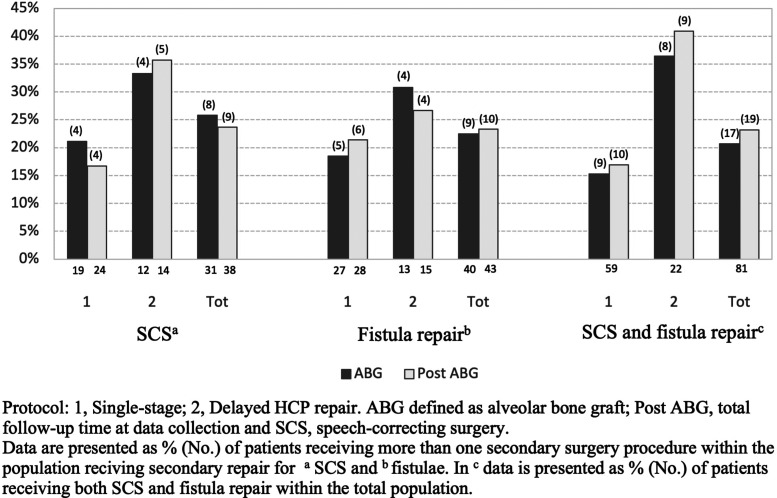
Multiple secondary surgery procedures.

Fistulas were mainly closed by suturing the oral and nasal mucosa in separate layers. If needed, lateral incisions and resorbable collagen membranes were used.

### Statistical Analysis

SPSS Statistics for Macintosh, version 25.0 (IBM Corp.) was used for statistical analyses. Categorical comparisons were performed with χ^2^ and Fisher's exact test, and the results were presented as proportions (%). Since the study groups were small and the variables were skewed, comparison of continuous data and nonparametric values was performed with Mann–Whitney U and Kruskal–Wallis tests, and the results are presented as medians with interquartile ranges (IQR). Throughout the study, differences were considered significant at *P* < .05.

## Results

### Sample Description

Altogether 81 consecutive children were eligible for the study (Table 1), and 67 patients did not meet the inclusion criteria and were therefore excluded. The participants were born between 1990 and 2010 and diagnosed with total BCLP. Twenty-seven patients (33.3%) presented with Simonart's band. Fifty-nine patients (72.8%) underwent single-stage repair, while 22 (27.2%) underwent palate repair in a two-stage procedure with delayed hard palate closure. Mean age at bilateral ABG was 9.9 years (9.4-10.8 years). Of the procedures, 71.6% (n = 58) were performed as a single bilateral procedure, whereas the rest were performed as two separate procedures; no significant difference emerged between the two protocols (72.9% vs 68.2%) (χ^2^ = .174, df = 1, *P* = .677). Mean age at total follow-up (post-ABG) was 17.7 years (14.2-20.8 years).

### Surgical Protocol

The majority of the study population underwent lip repair at 4.1 months (3.4-5.2 months) combined with palate repair in a single-stage procedure at 10.6 months (9.0-11.9 months), while 22 patients (27.2%) underwent lip and soft palate repair at 4.1 months (3.6-4.6 months) combined with delayed hard palate closure at 19.1 months (15.5-25.9 months). Bardach's two-flap palatoplasty was the most utilized technique (50.8%) in the single-stage protocol. No important differences regarding population demographics emerged between the surgical protocols.

The majority (82.7%, n = 67) of the primary operations were performed by two experienced senior cleft surgeons (50.6%, n = 41 and 32.1%, n = 26) who performed both protocols in a similar manner (χ^2^ = .386, df = 1, *P* = .535).

**Table 1.** Population Demographics.

	Single-stage	Two-stage	*P*
No. of patients	59	22	
Gender (male)	44 (74.6%)	17 (77.3%)	.804
Birth weight, grams	3460 (2840.0-3935.0)	3935 (2945.0-3750.0)	.889
Gestational age, weeks	39 (38.5-40.5)	39 (38.0-40.0)	.642
Age at lip repair, weeks	4.1 (3.4-5.2)	4.1 (3.6-4.6)	.975
Age at first palatoplasty, months	10.6 (9.0-11.9)	4.1 (3.7-5.5)	<.001
Age at second palatoplasty, months	N/A	19.1 (15.5-25.9)	
Age at ABG, years	9.9 (9.4-10.8)	9.9 (9.2-10.6)	.441
Post-ABG follow-up, years	18.0 (15.0-21.4)	16.6 (13.2-19.1)	.470

Categorical data is presented as n (%) while continuous data as median (interquartile range, IQR).

Post-ABG follow-up, total follow-up time at data collection.

Abbreviations: ABG, alveolar bone grafting; N/A, not applicable.

### Surgical Burden of Care

By the time the bilateral ABG had been performed, 54 patients (66.7%) had undergone secondary surgery on the palate and 29 (35.8%) had several procedures performed (SCS and/or fistula repair) (Figure 2). More precisely, 31 patients (38.3%) had undergone SCS due to VPI, while 40 patients (49.4%) had repair of fistulas, 24 (29.6%) of which were performed prior to ABG. At the end of the follow-up period (post-ABG), 60 patients (74.1%) had secondary procedures performed, while 34 (41.9%) had undergone several secondary procedures. SCS was performed on 38 patients (46.9%) and 43 (53.1%) underwent fistula repair. (Table 2) By the time of total follow-up (post-ABG), 27 patients (33.3%) had undergone orthognathic surgery (LeFort 1 osteotomy n = 25, bimaxillary osteotomy n = 1, segmental osteotomy n = 1) due to maxillary hypoplasia at a median age of 17.6 years (16.3-19.2 years). After osteotomy, 4 patients (4.9%) had their first SCS performed.

**Table 2.** Incidence of Speech-Correcting Surgeries (SCS) and Fistula Repair at ABG and Post-ABG in BCLP.

		SCS No. of patients (%)				Fistula No. of patients (%)					
Protocol	No. of patients (%)	ABG	* P*	Post-ABG	* P*	Pre-ABG	* P*	ABG	* P*	Post-ABG	* P*
**Single-stage**	**59**	**19 (32.2)**	.066^a^	**24 (40.7)**	.066^a^	**12 (20.3)**	.003^a^	**27 (45.8)**	.286^a^	**28 (47.5)**	.195^a^
Two-flap	30	9 (30.0)	.730^b^	11 (36.7)	.433^b^	5 (16.7)	.347^b^	14 (46.7)	.543^b^	14 (46.7)	.292^b^
Langenbeck	13	6 (46.2)		6 (46.2)		5 (38.5)		6 (46.2)		6 (46.2)	
Minimal incision	8	2 (25.0)		5 (62.5)		N/A		2 (25.0)		2 (25.0)	
V-W-K	8	2 (25.5)		2 (25.0)		1 (12.5)		5 (62.5)		6 (75.0)	
**Two stage**	**22**	**12 (54.5)**		**14 (63.6)**		**12 (54.5)**		**13 (59.1)**		**15 (68.2)**	
Total	81	31 (38.3)		38 (46.9)		24 (29.6)		40 (49.4)		43 (53.1)	

Categorical data is presented as n (%).

^a^ Comparison of the 2 surgical protocols.

^b^ Comparison of the single-stage techniques.

Post-ABG, SCS, and fistula repair are performed at total follow-up (data collection).

Abbreviations: ABG, alveolar bone grafting; BCLP, bilateral cleft lip and palate; Minimal incision, Mendoza's technique; Pre-ABG, fistula repair performed before ABG; Two-flap, Bardach's technique; V-W-K, Veau-Wardill-Kilner technique.

### SCS and Fistula Repair

The surgical protocols and outcomes are presented in Table 2. The single-stage protocol was associated with an overall lower rate of secondary surgeries than the two-stage protocol (69.5% vs 86.4%), regarding both SCS and fistula repair rates prior to ABG, although not statistically significant (χ^2^ = 2.375, df = 1, *P* = .123). By the time of ABG, the minimal incision, V-W-K, and two-flap technique presented with the lowest SCS rates (25.0%, 25.0%, and 30.0%, respectively) in the single-stage protocol, although compared with the two-stage protocol (54.5%) no significant differences were observed (*P* = .226, *P* = .101, and χ^2^ = 3.176, df = 1, *P* = .075). Prior to ABG the median age at first SCS were 5.7 (5.1-6.7) years, with corresponding 5.9 (5.2-8.4) years at the total follow-up. The most employed SCS technique was Furlow Z-plasty (Table 3).

**Table 3.** Surgical Method and age at First SCS.

			SCS No. of patients (%)		
Protocol	Time point	No. of patients	Pharyngeal flap	Furlow	Other
Single-stage	ABG	19	6 (31.6)	11 (57.9)	2 (10.5)
	Post-ABG	24	6 (25.0)	16 (66.7)	2 (8.3)
Two-stage	ABG	12	4 (33.3)	7 (58.3)	1 (8.3)
	Post-ABG	14	4 (28.6)	9 (64.3)	1 (7.1)

Post-ABG and SCS is performed at total follow-up (data collection).

Abbreviations: ABG, alveolar bone grafting; SCS: speech-correcting surgery.

Before ABG, the single-stage protocol had a significantly lower fistula repair rate (20.3% vs 54.5%) (χ^2^ = 8.993, df = 1, *P* = .003) than the two-stage protocol. More specifically, the two-flap technique had significantly lower rates (16.7%) than the two-stage procedure (χ^2^ = 8.276, df = 1, *P* = .004). However, at the time of ABG, the No. of fistulas repaired in the palate in the single-stage protocol doubled, while the rates for the two-stage protocol almost stayed the same, this difference between the protocols was found significant (χ^2^ = 8.376, df = 1, *P* = .004). A significant difference (*P* = .041) emerged between the surgical protocols regarding the location of the repaired fistulas. The one-stage protocol had a high representation of corrected fistulas in the hard palate (93.3%) (Pittsburgh IV-V), especially anterior fistulas (63%) (Pittsburgh V), whereas the two-stage protocol had a more evenly distributed pattern of repaired fistulas in the palate. One-third of the fistulas were connected to a perialveolar fistula (Table 4).

**Table 4.** Location of Repaired Fistulas by the Time of ABG.

	Pittsburgh location							
Protocol	No. of patients (%)	II	III	IV	V	* P*	Connection to perialveolar fistula	* P*
**Single-stage**	**30**	**2 (6.7)**	**0 (0.0)**	**9 (30.0)**	**19 (63.3)**	.041^a^	**13 (43.3)**	.074^a^
Two-flap	15	2 (13.3)	N/A	1 (6.7)	12 (80.0)	.010^b^	7 (46.7)	.004^b^
Langenbeck	6	N/A	N/A	5 (83.3)	1 (16.7)		N/A	
Minimal incision	4	N/A	N/A	2 (50.0)	2 (50.0)		1 (25.0)	
V-W-K	5	N/A	N/A	1 (20.0)	4 (80.0)		5 (100.0)	
**Two-stage**	**17**	**1 (5.9)**	**4 (23.5)**	**5 (29.4)**	**7 (41.2)**		**3 (17.6)**	
Total	47	3 (6.4)	4 (8.5)	14 (29.8)	26 (55.3)		16 (34.0)	

Six patients had 2 fistulas repaired at different locations in the palate. Categorical data is presented as n (%).

^a^ Comparison of the 3 surgical protocols.

^b^ Comparison of the single-stage techniques.

Abbreviations: ABG, alveolar bone grafting; Minimal incision, Mendoza's technique; N/A, not applicable; SCS, speech-correcting surgery; two-flap, Bardach's technique; V-W-K, Veau-Wardill-Kilner technique.

There was a significant difference in SCS rates prior to ABG between the two surgeons performing the majority of the primary repairs (51.2% vs 14.4%) (χ^2^ = 8.735, df = 1, *P* = .033). The surgeon with higher SCS rates performed 93.5% of the SCS procedures. In contrast, no significant difference was noted in fistula repair rates between the surgeons (48.8% vs 53.8%) (χ^2^ = .163, df = 1).

## Discussion

Here, we report the long-term need for secondary surgeries, defined as SCS and fistula repair, in a non-syndromic BCLP population treated at a single cleft center. We also compare two surgical protocols utilized over the years by assessing complication rates, defined as a quality measurement of the primary repair.

In general, little has been reported regarding the surgical management of BCLP and its long-term outcomes, with the literature tending to focus on the more common cleft types. However, when reported, BCLP is often associated with poorer speech outcomes and higher complication rates than the other less extensive cleft types.^15–20^ Comparing our outcomes with two earlier studies by the same cleft center, reporting long-term surgical outcomes in isolated cleft palates^12^ and UCLP,^5^ as expected, the same trend of poorer outcomes in BCLP was evident. Our results showed somewhat higher rates for SCS and fistula repair than previous reports on VPI, fistula occurrence, and secondary repair rates in BCLP. This may partly be explained by the much shorter follow-up in the majority of earlier studies.^15,18,19,21^ Particularly notable was the significantly high rates of fistula repair at ABG. Similar observations in BCLP are in line with Shankar et al.^22^ reporting late fistula rates at ABG as high as 76% while David et al.^2^ reported 79%, since a large proportion of fistulas remain undiagnosed prior to the intraoperative examination at ABG.^22^

Comparisons between surgical protocols and techniques utilized at primary repair in BCLP are limited,^21^ and the studies usually have small caseloads^20–22^ and little insight into surgical management or long-term outcome. Generally, similar surgical principles as for UCLP cleft repair are utilized for BCLP. However, considering the variable severity of the cleft width and protrusion of the premaxilla causing marked and complex anatomical variations in the bilateral cleft, one technique or protocol is hardly suitable for all cleft types. Individual anatomical variations in techniques are often necessary, and an experienced and highly skilled surgeon is crucial.

As often with cleft studies, only small, if any, differences emerge between different surgical protocols and techniques. Although this is a result of a wide range of impacting factors, not uncommonly, this is caused by sample sizes that are inadequate to obtain a significant difference. Correspondingly, in the present study, although the single-stage protocol seemed to have slightly inferior long-term results in our surgeon’s hands, only a few significant differences were noted between the two protocols. This might, however, be partly explained by the overall small caseload, particularly in the two-stage protocol, as well as the implementation of an unfamiliar surgical protocol for the surgeons. As described in the SC study, long learning curves reflected struggles in adopting new surgical techniques in low caseloads.^11^

Nevertheless, a few differences were noted between the protocols. Corresponding to the SC study,^11^ the single-stage approach had significantly (*P* = .03) lower fistula repair rates prior to ABG than the two-stage approach. Particularly, the two-flap technique presented with low fistula repair prior to ABG, and compared with the two-stage procedure a significant difference emerged. Comparable outcomes were present in an earlier study of the same center investigating long-term results in UCLP^5^ However, the single-stage procedure was associated with a high need for fistula closure at ABG, especially in the anterior part, at the level of the incisive foramen (at the junction of the primary and secondary palates), often linked to perialveolar fistulas. The same phenomenon was also noted in David et al.’s^2^ study, assessing children with BCLP that had received single-staged two-flap primary palatoplasty at 12 months of age. They reported a 79% rate of anterior fistulas, which most of them were repaired at ABG. The reason behind these anterior fistulas may partly be due to presurgical orthodontic treatment and widening of the anterior palate, which may reveal fistulas in this area. In the present study, this phenomenon was particularly notable in the V-W-K and the Bardach two-flap approach, which shows the difficulty of closing the anterior palate, even with these methods. According to the senior author, especially challenging was to stretch the mucoperiosteal vomer flaps to reach the most anterior part of the cleft and premaxilla, often leading to a one-layer closure in this area, which for a variety of reasons is inadequate for a secure closure. Patients with cleft lip and palate have a high representation of fistulas in the hard palate (IV).^14,22,23^ However, we noted a high presentation of anterior (V) fistulas, although this may be explained by the fact that the fistulas were localized once, according to their most anterior position, whereby some IV fistulas may be counted as V fistulas. There is a general ambiguity regarding fistula nomenclature and given the retrospective nature of the vast majority of studies, this inevitably leads to study biases. These, in turn, further limit meaningful comparison of results across institutions. A comparable trend of high fistula repair rates at ABG in the single-stage approach was noted in an earlier UCLP study at our center.^5^ And, as already stated, although large anterior fistulas undoubtedly are a challenging surgical dilemma,^24^ we believe that the majority of these anterior fistulas closed at ABG represent smaller, less significant, and less symptomatic fistulas than larger anterior fistulas or the more posteriorly located fistulas that require closure in a separate operation prior to ABG. This statement is in line with Andersson et al.^25^ who reported that posterior fistulas are more often associated with VPI. Moreover, fistulas requiring early repair have previously been described as particularly challenging since they are more likely to develop recurrently.^22^ In contrast to the single-stage protocol, 12 of 13 patients (92.3%) in the two-stage procedure underwent fistula closure prior to ABG as a separate procedure. Of these fistulas, 33% (n = 5) were Pittsburgh V and IV fistulas, whereas 26.7% (n = 4) were III fistulas and 6.1% (n = 1) II fistulas.

Among the total population, 46.9% (n = 38) underwent SCS during the full follow-up period of which 4 patients received repair following orthognathic surgery. While the amount seems high compared to the previous literature,^15,16,18,26^ cross-comparisons are difficult because of the aforementioned reasons. Considering the known associated obstruction risk with pharyngeal flaps^27^ which still today universally represent the most common type of VPI surgery, followed by sphincter pharyngoplasty, the Furlow Z-plasty is yet not as well established.^28^ However, in our opinion, Furlow Z-plasty is not only an effective way of treatment but also a safe procedure, therefore it has served as the primary SCS choice at our unit for over 2 decades and might describe the potential lower threshold for SCS and higher surgery rates compared to initial pharyngeal flap procedures. However, our results are comparable with the long-term follow-up of David et al.,^2^ which reported a VPI surgery incidence of 42.1% within a BCLP population. Regarding SCS surgery rates, no significant differences emerged between the two surgical protocols, although we found somewhat better speech outcomes with less need for SCS in the single-stage protocol than in the two-stage approach (32.2% vs 54.5%). Likewise, no significant differences were present between the single-stage techniques or among the separate single-stage techniques compared with the two-stage approach. Although V-W-K presented the lowest SCS rates (Table 2), attention was drawn to the favorable results of the Bardach two-flap technique, although this technique is often utilized in particularly wide and challenging clefts. The two-flap technique preserves the anatomy of the cleft. Releasing the muscle of the posterior edge of the hard palate, the anatomical dissection of the muscle, and the elevation of the mucoperiosteal flaps seem to facilitate the release of the tethering soft tissue structures of the soft palate, which in turn allows retroposition of the soft palate.

As surgical treatment protocols have evolved over the years, they have been challenged by controversies concerning speech and growth of the maxillary structures.^29^ To address this issue, two-stage protocols have been developed by delaying either the hard palate or the soft palate closure. Yet, there is some disagreement regarding the timing. Some authors are concerned about early palatoplasty's impact on growth of the maxillary structures. However, emerging evidence suggests that children are not capable of developing normal speech patterns if hard palate repair is delayed by years,^30–33^ advocating early cleft repair by 12 months of age.^34,35^ Moreover, in the SC study trial 1, comparing two-stage palatoplasty with hard palate closure at 12 months and 36 months, the latter was associated with poorer consonant proficiency and higher need for speech therapy visits at 5 years of age, although no difference in velopharyngeal competency and hypernasality was noted nor in VPI surgery rates at 5 or 9 years.^11,36^

Recently, increasing attention has been drawn to the encouraging reported outcomes of two-stage closure with early hard palate closure and delayed soft palate repair in UCLP. This is especially due to the favorable condition at soft palate repair, with a reduction in cleft width^37^ that allows a repair with less tension, resulting in particularly low fistula rates. However, variable opinions have been presented regarding speech outcome; some authors are concerned about scar contracture resulting in shorter palates,^38^^,39^ while others compensate for this by applying lengthening Z-plasty at soft palate repair.^40^ Early hard palate repair has also been employed at our clinic in both UCLP and BCLP since 2009, but due to the yet short follow-up and low caseload, this protocol was not included here. However, the results would be of interest for this study. Reflecting surgical burden of care and used to compare long-term outcomes of surgical protocols, maxillary growth is also an important outcome measure for the need for orthognathic surgery, and overall esthetical outcome. In this study, osteotomy rates were somewhat lower than in a previous report from our center.^41^ However, this is mainly due to the still short follow-up period since most operations are performed in late adolescence. In general, evidence regarding how different surgical protocols affect maxillary growth in BCLP is limited by the small caseloads,^42^ and therefore, we intend to report on the impact of these protocols on maxillary growth and dental arch relationships in the future.

Strengths of this study include the large comprehensive data on a BCLP population collected at a single center where the patients received frequent management in a multidisciplinary setting with a long-term follow-up. The study was limited by its retrospective design and the inherent potential for bias. The authors were restricted by the quality of the operation records, which may have caused interpretation errors while assessing fistula occurrence and surgical techniques. As is common in surgery, modifications to the surgical techniques may have been made by the surgeons in adapting to the severity and anatomy of individual clefts. The comparison of the two protocols was not ideal in view of the lower caseload of the two-stage procedure. Although the majority of the surgeries were performed by experienced cleft surgeons, we noted a significant difference between the surgeons in terms of SCS rates. However, not only were there no differences in fistula repair rates between the surgeons, but the surgeon with higher SCS rates performed most of the SCS procedures. As such, our aim was not to compare surgeons, although surgeons’ skill has a well-known impact on surgical outcome. To draw conclusions regarding surgeons’ skill, more detailed studies of cleft severity and speech are warranted.

## Conclusions

BCLP constitutes a challenge for the whole cleft team and has a high burden of care in terms of secondary surgeries, defined as SCS and fistula repair. Limited information is available on the impact of different surgical protocols on BCLP speech, fistulas, and maxillary growth, particularly from a long-term perspective. In our hands, the single-stage approach seems to offer better—albeit not significant—speech outcomes than the short delayed hard palate closure and entails less separate fistula repair operations before ABG. Overall, making definitive conclusions regarding surgical protocols and techniques is challenging due to a general lack of standardized outcome measurements and classifications between institutions, which in turn makes exact size analysis impossible. Finally, as stated in the SC study (Shaw and Semb, 2017), the question remains of whether a single protocol or specific surgical method can outweigh the importance of an experienced surgeon mastering a familiar method.
